# Real-world setting comparison of bridging therapy versus direct mechanical thrombectomy for acute ischemic stroke: A meta-analysis

**DOI:** 10.1016/j.clinsp.2024.100394

**Published:** 2024-05-30

**Authors:** Bin Qin, Tao Wei, Wen Gao, Hui-xun Qin, Yu-Ming Liang, Cheng Qin, Hong Chen, Ming-Xiu Yang

**Affiliations:** a Department of Neurology, Liuzhou People's Hospital, Liuzhou, Guangxi, China; b Liuzhou Key Laboratory of Epilepsy Prevention and Research, Liuzhou, Guangxi, China; c Department of Neurology, The First Affiliated Hospital of Guangxi Medical University, Nanning, Guangxi, China

**Keywords:** Acute ischaemic stroke, Large-vessel occlusion, Intravenous thrombolysis, Bridging therapy, Real-world evidence, Meta-analysis

## Abstract

**Background and purpose:**

Intravenous Thrombolysis (IVT) prior to Mechanical Thrombectomy (MT) for Acute Ischaemic Stroke (AIS) due to Large-Vessel Occlusion (LVO) remains controversial. Therefore, the authors performed a meta-analysis of the available real-world evidence focusing on the efficacy and safety of Bridging Therapy (BT) compared with direct MT in patients with AIS due to LVO.

**Methods:**

Four databases were searched until 01 February 2023. Retrospective and prospective studies from nationwide or health organization registry databases that compared the clinical outcomes of BT and direct MT were included. Odds Ratios (ORs) and 95 % Confidence Intervals (CIs) for efficacy and safety outcomes were pooled using a random-effects model.

**Results:**

Of the 12 studies, 86,695 patients were included. In patients with AIS due to LVO, BT group was associated with higher odds of achieving excellent functional outcome (modified Rankin Scale score 0–1) at 90 days (OR = 1.48, 95 % CI 1.25–1.75), favorable discharge disposition (to the home with or without services) (OR = 1.33, 95 % CI 1.29–1.38), and decreased mortality at 90 days (OR = 0.62, 95 % CI 0.56–0.70), as compared with the direct MT group. In addition, the risk of symptomatic intracranial hemorrhage did not increase significantly in the BT group.

**Conclusion:**

The present meta-analysis indicates that BT was associated with favorable outcomes in patients with AIS due to LVO. These findings support the current practice in a real-world setting and strengthen their validity. For patients eligible for both IVT and MT, BT remains the standard treatment until more data are available.

## Highlights

• Acute ischaemic stroke is becoming a major public health concern.
• The nationwide or health organization registry databases were included.
• Bridging therapy remains the standard treatment until more data are available.

## Introduction

Stroke is becoming increasingly significant as a major public health concern. It continues to hold its position as the second highest cause of death globally, and when considering death and disability together, it ranks as the third highest cause worldwide. In terms of the number of cases, the burden of stroke has noticeably risen, as evidenced by the latest Global Burden of Diseases stroke burden estimates for 2019.^1^ Approximately 70 % of strokes worldwide occur as ischaemic strokes, and the proportion in the United States is higher, at about 85 %–87 %; of which, Large-Vessel Occlusion (LVO) accounts for half of them.^2^ Patients with Acute Ischaemic Stroke (AIS) caused by LVO usually have bigger infarct sizes, more disabling symptoms, and high mortality rates and poor outcomes.^3^ Fortunately, reperfusion therapies have improved the clinical outcomes of many patients with AIS, preventing death and disability. However, the treatment of patients with AIS depends on the time since stroke onset, the severity of neurologic deficit, and the results of neuroimaging. Treatment options for AIS need to be understood in order to ensure prompt treatment and improve clinical outcomes.

Intravenous Thrombolysis (IVT) has been proven to be safe and effective in improving the clinical prognosis of patients with AIS. However, by incorporating its narrow therapeutic time window, there are a number of limitations that prevent its wider development and use.^4^ The low rate of early reperfusion among patients with LVO also limits IVT's efficacy.^4^ Thus, for patients with AIS caused by an anterior circulatory LVO, Mechanical Thrombectomy (MT) has become the standard treatment.^5^ One proposal is that administering IVT before MT (known as Bridging Therapy [BT]) could decrease the duration of successful MT by altering the characteristics of the blood clot, enhancing its responsiveness to mechanical intervention, and eliminating any remaining thrombotic material. This suggestion takes into account the distinctions between IVT and MT regarding procedures, time frame, and canalization mechanism. Therefore, for patients with AIS who are eligible for both IVT and MT, current guidelines recommend BT. However, IVT has inherent unfavorable effects despite its substantial efficacy as a reperfusion strategy. The use of IVT may delay the start of MT and increase the risk of Symptomatic Intracranial Haemorrhage (sICH), and IVT can also lead to distal migration due to thrombus fragmentation as well as higher procedure costs of procedures.^6^ In light of these opposing factors, investigators have questioned whether pre-treatment with IVT is beneficial in the long run.

Over several years, meta-analyses of observational studies comparing BT with direct MT have suggested that BT has a greater benefit.^7^, ^8^, ^9^ Nevertheless, pooling observational study datasets without properly matching statistical techniques may produce flawed conclusions. On the other hand, several groups have performed Randomised Controlled Trials (RCTs) to examine the efficacy and safety of BT in patients with LVO compared with direct MT. The results of these RCTs and their meta-analyses showed that direct MT had comparable consequences to BT in terms of efficacy and safety outcomes for patients with AIS due to LVO.^4^^,^^10^, ^11^, ^12^, ^13^, ^14^ which was inconsistent with the aforementioned meta-analyses of observational studies. A recent individual participant data meta-analysis of RCTs also did not establish non-inferiority of direct MT compared with BT.^15^ However, several methodological factors confound the interpretation of the results, including not meeting the pre-specified margins of noninferiority and terminating early. Moreover, it is important to remember that RCTs are limited in their ability to generalize to larger and often more representative populations of patients, healthcare providers, and settings where concomitant disorders, medications, and adherence to treatment may differ significantly.^16^, ^17^, ^18^ In addition to inappropriate physician decisions, lack of professional equipment, and other factors might also contribute to the reduction of the therapeutic effect of MT in real-world practice.^19^ Real-world evidence, such as electronic health records, claims data, and disease registries, complements clinical trial evidence derived from RCTs.^16^ More recently, several reports were published comparing the safety and efficacy of BT with direct MT in real-world settings, such as national patient samples, multinational, and nationwide registries.^20^, ^21^, ^22^, ^23^ However, these reports differ significantly in terms of data sources and methods of analysis.

Therefore, the authors performed a meta-analysis of the available real-world evidence from nationwide or health organization registry databases focusing on the efficacy and safety of BT compared with direct MT in patients with AIS due to LVO.

## Methods

The authors followed the Preferred Reporting Items for Systematic Reviews and Meta-Analysis (PRISMA) statement guidelines when writing this report, and the study was registered in the PROSPERO database (CRD42023421973).

### Patient and public involvement

There was no patient or public involvement, and ethical approval was not required for this study.

### Search strategy and eligibility criteria

The authors searched PubMed, Cochrane Library, Embase, and Web of Science until 01 February 2023 using the terms “stroke” AND “thrombectomy OR endovascular” AND “thrombolysis OR alteplase OR bridging therapy” AND “real world” OR “observational” OR “registry” OR “cohort” (Table S1 in the Supplementary File). The references of previous reviews and included studies of this meta-analysis were manually screened to avoid missing any eligible studies that were not previously identified. The authors included all studies satisfying the following criteria: (1) Observational studies that compared BT with direct MT in adult patients with AIS due to LVO, and to ensure that the highest quality datasets were included, only nationwide or health organization registry databases were eligible.^16^ (2) At least one outcome of interest must have been reported in the included studies; and (3) When the included studies used the same data source with overlapping study periods, the authors only included the study with the longest study period unless the study periods did not overlap or unless the study included data from another source. Studies were excluded if they (1) were post hoc analyses of RCTs and (2) were case reports, reviews, or studies with incomplete data.

### Study selection, data extraction, study outcomes, and assessment of bias

Two investigators (BQ and WG) independently screened the titles and abstracts, and full-text articles were reviewed and evaluated. Any discrepancies or uncertainties were resolved through consensus or discussion with a third investigator (MXY). Two investigators (BQ and TW) independently extracted the following data using a standardized form: first author, publication date, study location, demographic characteristics, vessel occlusion sites, functional outcomes, sICH, mortality, and other detailed characteristics of the included studies. Disagreements were resolved by consensus or discussion with a third investigator (MXY).

The primary efficacy outcomes were the proportion of patients who achieved excellent functional outcomes (modified Rankin Scale [mRS] score 0–1) at 90 days and favorable discharge disposition (to the home with or without services). Secondary efficacy outcomes were the proportion of patients who achieved favorable functional outcomes (mRS score 0–2) at 90 days and successful reperfusion on postprocedural angiography (defined as Thrombolysis in Cerebral Infarction score ≥2b). Safety outcomes were based on the rates of sICH and mortality at 90 days. sICH was defined according to the Heidelberg Bleeding Classification, Safe Implementation of Thrombolysis in Stroke-Monitoring Study (SITS-MOST) criteria, European Cooperative Acute Stroke Study II criteria (ECASS-II), and ECASS-III criteria.

The authors used the Risk of Bias in Non-randomised Studies of Interventions (ROBINS-I) tool.^24^ to assess the quality of the studies included. The tool evaluates the specific outcomes of each study in seven domains related to the intervention being studied. The level of bias risk in each domain was classified as low, moderate, serious, critical, or no information. Two investigators (BQ and TW) independently evaluated each eligible study, and any disagreements were resolved through discussion with a third investigator (MXY).

### Data analysis

Pooled Odds Ratios (ORs) with 95 % Confidence Intervals (CIs) were calculated for each outcome between patients receiving BT and those receiving direct MT using random-effects models. For the primary efficacy and safety outcomes (excellent functional outcome at 90 days, favorable discharge disposition, rates of sICH, and mortality at 90 days), the authors also calculated the pooled adjusted OR (multiple regression or matching analyses) when reported. The authors used the logarithmic-adjusted OR and the corresponding standard errors to calculate the pooled adjusted OR in a random-effects analysis. Logarithmic ORs were calculated with lnOR; standard errors were calculated using (upper CI – lower CI)/3.92. The heterogeneity between studies was evaluated using the p-value of the χ^2^ statistics. The I^2^ statistics were used to quantify the heterogeneity between studies. Mild, moderate, and high heterogeneity were identified as I^2^ values of around 25 %, 50 %, and 75 % respectively.^25^ In addition, the authors performed a sensitivity analysis by limiting the studies to those on AIS attributable to anterior circulation occlusion for the primary efficacy and safety outcomes. All statistical tests were two-sided, with a significance threshold of *p* ≤ 0.05. A funnel plot of the reported effect estimates was used to assess for risk of publication bias; in addition, egger's regression test was employed to evaluate the presence of publication bias.^25^ All data were analyzed with Review Manager (RevMan) (version 5.4; the Cochrane Collaboration, 2020).

## Results

### Study selection and characteristics

**Fig. 1:**
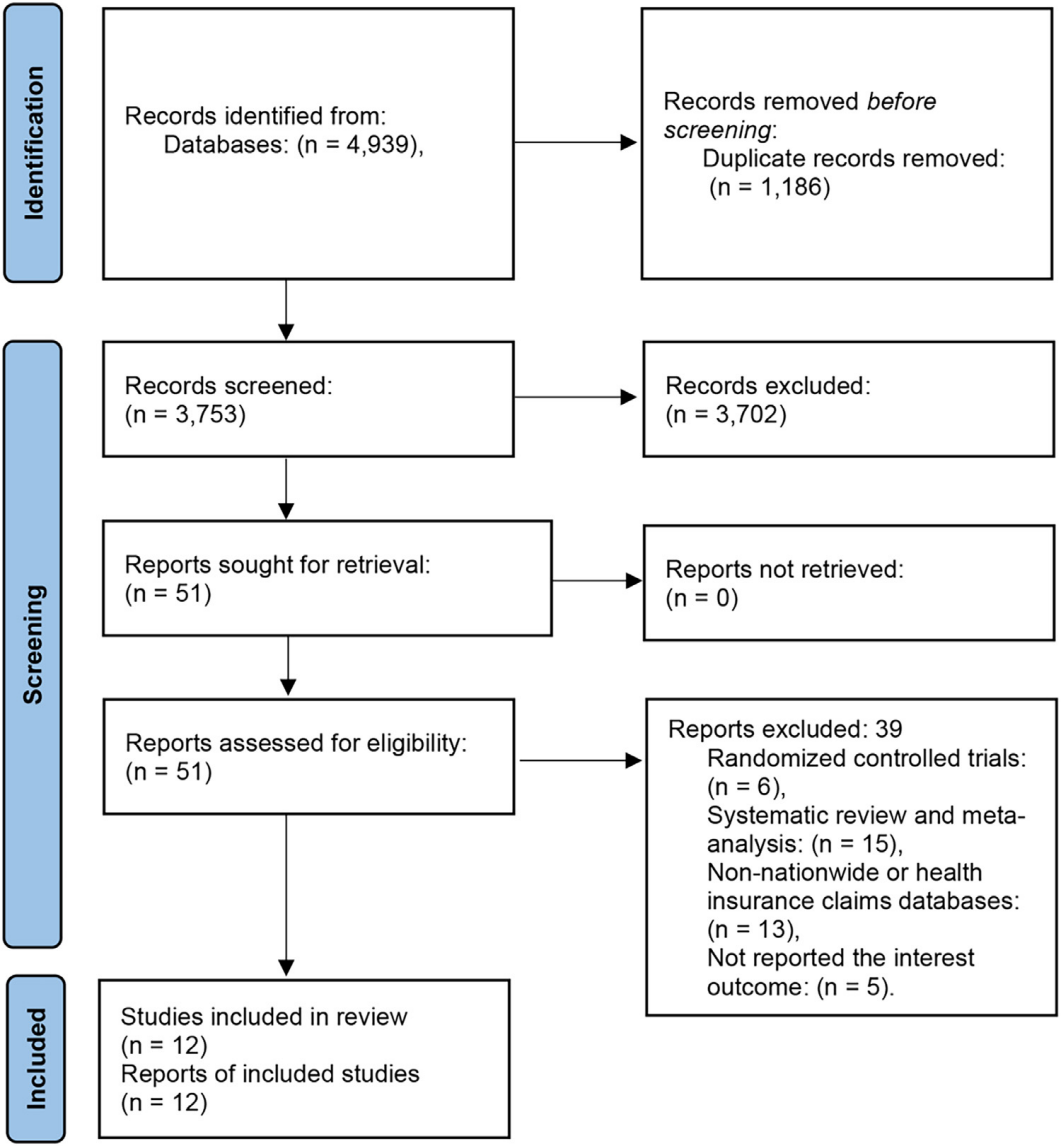
flowchart of the selection process in this meta-analysis.

A total of 4939 records were searched using electronic databases, of which 1186 were excluded because they were duplicates. After retrieving 51 articles for full-text review, 39 articles were excluded because of inappropriate article types (6 RCTs and 15 systematic reviews and meta-analyses), no nationwide or health insurance claims databases, and no reported interest outcomes. Twelve studies were eventually included after qualitative and quantitative analysis, containing 86,695 patients.^20^, ^21^, ^22^, ^23^^,^^26^, ^27^, ^28^, ^29^, ^30^, ^31^, ^32^, ^33^ The PRISMA flowchart of the study inclusion process can be seen in Fig. 1.

The main characteristics of the studies included in this meta-analysis are summarised in Table 1. A total of 41,284 patients (47.6 %) in the BT group and 45,411 patients (52.4 %) in the direct MT group were included in the quantitative analysis. Mean/median age was similar between groups (68.5 years for BT and 69.7 years for direct MT). Female patients accounted for 50 % of cases. Most studies used alteplase as a thrombolytic agent, except for one study that used alteplase and Tenecteplase. The mean/median baseline NIHSS score ranged from 11 to 18 points and was predominantly 16 to 18 points. A total of 7/12 of the included studies included only patients with anterior circulation AIS.

**Table 1:** Details of studies included in the meta-analysis.

Author and year	Study duration	Countries	Data source	Study design	Occluded Vessel	BT					Direct MT			
						N° of patients	Age (year)^a^	Female	NIHSS at Admission^a^	IVT	N° of patients	Age (year)^a^	Female	NIHSS at admission^a^
Ahmed 2021	2014 to 2019	European Union, Norway and Iceland	Safe Implementation of Treatment in Stroke–International Stroke Thrombectomy Register	P	AC	3944	72 (62–80)	49.9 %	16 (11–20)	Alteplase	2406	74 (64–81)	48.9 %	16 (11–20)
Casetta 2019	2011 to 2015	Italy	The Italian Registry of Endovascular Stroke Treatments	P	AC	635	67.6 (14.6)	50.7 %	18 (14–21)	Alteplase (0.9 mg/kg body weight, maximum 90 mg)	513	68.8 (13.1)	51.1 %	18 (14–22)
Chalos 2019	2014 to 2016	Netherlands	Multicenter Randomised Controlled Trial of Endovascular Treatment for Acute Ischemic Stroke in the Netherlands Registry	P	AC	1161	70 (59–79)	46.0 %	16 (11–20)	Alteplase (0.9 mg/kg body weight)	324	72 (63–80)	47.0 %	17 (13–20)
Di Maria 2018	2012 to 2016	France	Endovascular Treatment in Ischemic Stroke registry	P	AC	976	67.2 (15.0)	45.7 %	17 (11–20)	NA	531	67.6 (15.1)	48.8 %	16 (11–21)
Dicpinigaitis 2022	2015 to 2018	United States	The National Inpatient Sample (inpatient care databases in the United States).	R	AC	19,735	68.9 (0.2)^b^	50.9 %	NA	Alteplase	28,790	69.7 (0.2)^b^	53.0 %	NA
Geng 2021	2018 to 2019	China	China Stroke Prevention Project Committee– Direct Endovascular Thrombectomy and Bridging Thrombolysis registry	R	NA	2069	68 (58–76)	40 %	16 (12–20)	Alteplase	5605	67 (58–75)	40 %	16 (12–21)
Le Floch 2022	2015 to 2022	France	Endovascular Treatment in Ischemic Stroke registry	R	AC	570	69.5 (15.4)	48.6 %	11 (7–17)	Alteplase or Tenecteplase	562	71.8 (13.9)	50.0 %	12 (8–18)
Leker 2018	2014 to 2016	Israel	National Acute Stroke Israeli Survey of Patients Undergoing Revascularization	P	AC	159	68.1 (1.0)	43.0 %	16.0 (13–19)	Alteplase	111	67.4 (15.5)	48.0 %	16 (12–20)
Minnerup 2016	2012 to 2013	Germany	Register on Revascularization in Ischemic Stroke Patients	P	AC+PC	603	68.3 (13.7)	49.6 %	15.1 (6.4)	Alteplase	504	68.7 (14.7)	52.0 %	14.3 (6.7)
Park 2017	2008 to 2013	Korea	Clinical Research Center for Stroke-5th division registry	R	AC+PC	458	68 (12)	43 %	15 (11–19)	Alteplase (0.6/0.9 mg/kg body weight)	181	69 (12)	43 %	15 (10–18.5)
Smith 2022	2019 to 2020	United States	Get with the Guidelines-Stroke registry	R	AC+PC	10,548	70.0 (59.0–81.0)	49.1 %	16.0 (11.0–21.0)	Alteplase	5284	74.0 (63.0–83.0)	52.2 %	17.0 (11.0–22.0)
Tong 2021	2017 to 2019	China	Endovascular Treatment Key Technique and Emergency Workflow Improvement of Acute Ischemic Stroke registry	P	AC+PC	426	64 (55–72)	37.6 %	16 (11–20)	Alteplase (0.9 mg/kg body weight, maximum 90 mg)	600	66 (55–74)	34.7 %	17 (13–22)

a Mean ± Standard Deviation (SD) or median (Interquartile Range [IQR]) reported.

b Mean ± standard error of the mean AC, Anterior Circulation; BT, Bridging Therapy; IVT, Intravenous Thrombolysis; MT, Mechanical Thrombectomy; NA, Not Available; NIHSS, National Institutes of Health Stroke Scale; P, Prospective Study; PC, Posterior Circulation; R, Retrospective Study.

### Risk of bias and publication bias

The risk of bias for each individual study using the ROBINS-I tool and across all studies was an overall variable as seen in Table S2 in the Supplementary File. Most studies had a low-to-moderate risk of bias. Visual inspection of the funnel plots and calculation of the Egger test results should not be reported because no more than 10 studies reported each main outcome.

### Primary efficacy outcomes

**Fig. 2:**
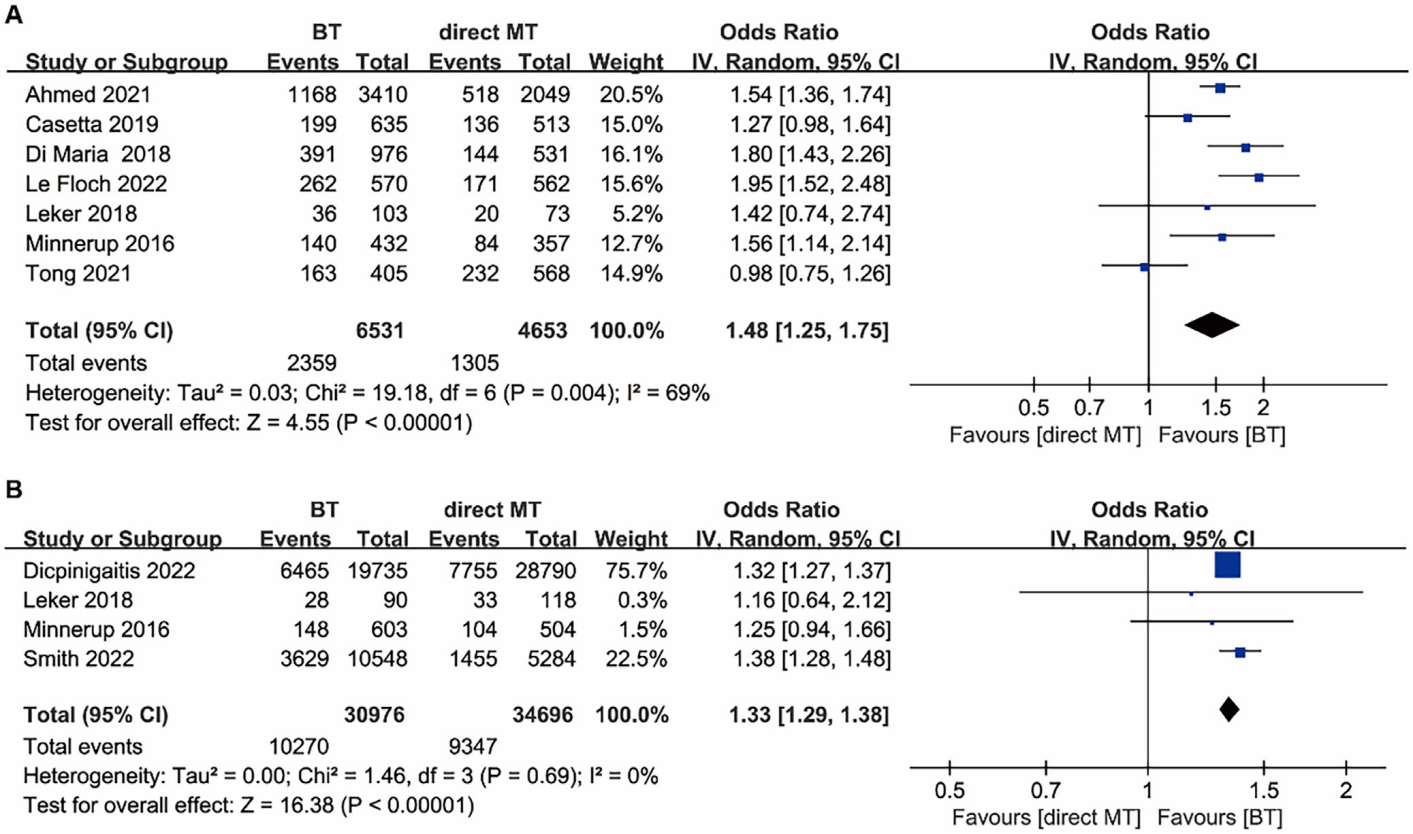
Forest plot of the odds ratios of primary efficacy outcomes in patients with acute ischaemic stroke: (A) Excellent functional outcome (mRS 0–1) at 90 days; (B) Favourable discharge disposition (to the home with or without services). BT, Bridging Therapy; CI, Confidence Interval; IV, Inverse Variance; MT, Mechanical Thrombectomy; mRS, Modified Rankin Scale.

Excellent functional outcome data were available in seven of the included studies. The BT group had statistically significant higher odds for excellent functional outcome (mRS score 0–1) at 90 days compared with the direct MT group (OR = 1.48, 95 % CI 1.25–1.75, *p* < 0.00001; Fig. 2A), with moderate heterogeneity (I^2^ = 69 %); and six included studies provided excellent functional outcome data of the adjusted effect estimates. Similarly, the BT group had statistically significant higher odds for excellent functional outcome of the adjusted effect estimates compared with the direct MT group (OR=1.28, 95 % CI 1.04–1.57, *p* = 0.02; Fig. S1A in the Supplementary File). The BT group also had higher odds for favorable discharge disposition (to the home with or without services) (OR = 1.33, 95 % CI 1.29–1.38, *p* < 0.00001; Fig. 2B), with no statistically significant heterogeneity (I^2^ = 0 %). The favorable discharge disposition of the adjusted effect estimates with the BT group was also higher than the direct MT group (OR = 1.27, 95 % CI 1.17–1.38, *p* < 0.00001; Fig. S1B in the Supplementary File).

### Safety outcomes

**Fig. 3:**
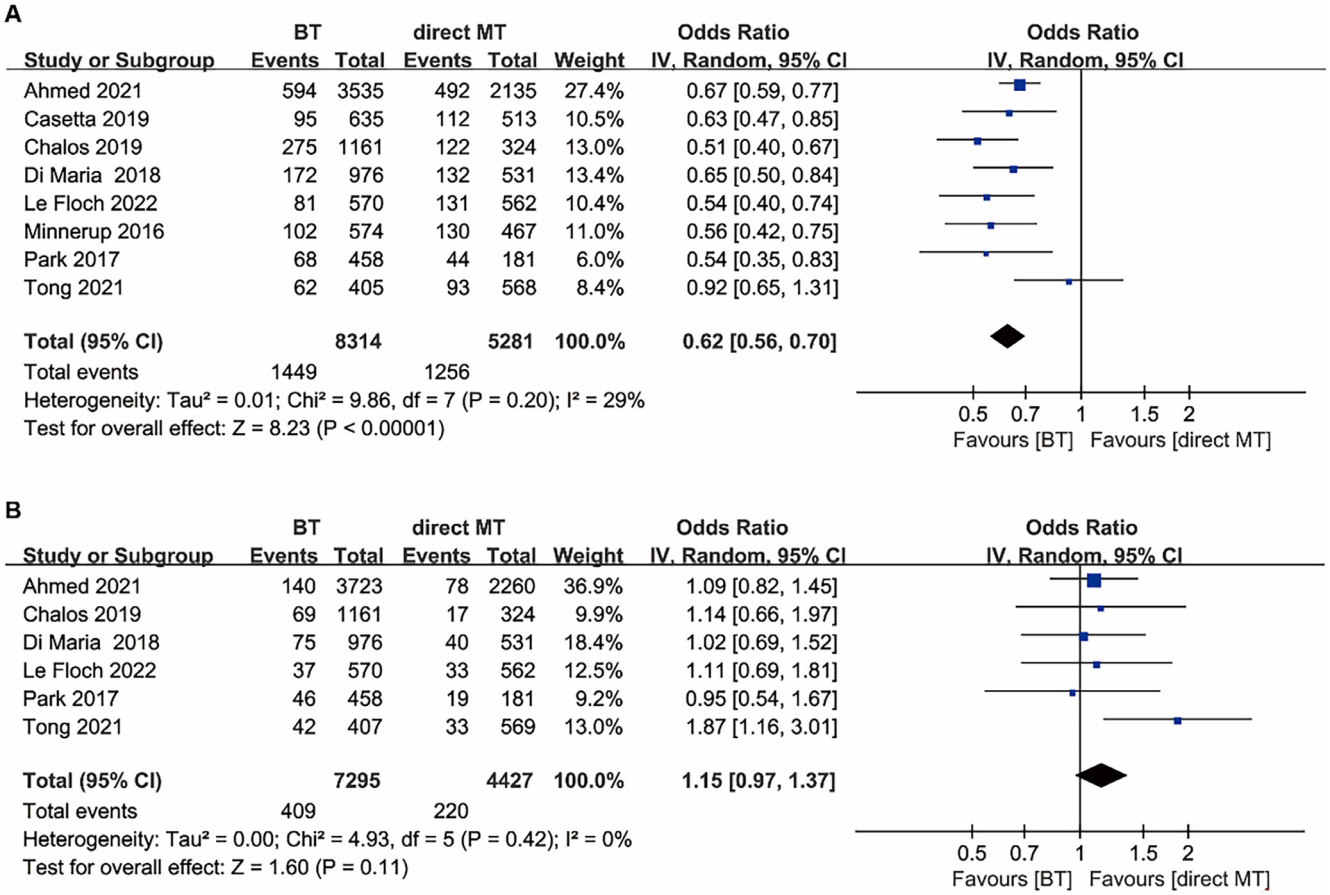
Forest plot of the odds ratios of the safety outcomes in patients with acute ischaemic stroke: (A) Mortality at 90 days; (B) Symptomatic intracranial hemorrhage. BT, Bridging Therapy; CI, Confidence Interval; IV, Inverse Variance; MT, Mechanical Thrombectomy.

Mortality at 90 days was available for eight included studies. Mortality at 90 days was lower in the BT group than the direct MT group (OR = 0.62, 95 % CI 0.56–0.70, *p* < 0.00001; Fig. 3A), with mild heterogeneity (I^2^ = 29 %), and the same result was achieved for the mortality of the adjusted effect estimates (OR = 0.73, 95 % CI 0.65–0.82, *p* < 0.00001; Fig. S2A in the Supplementary File). For rates for sICH, however, the BT group was not significantly different compared with the direct MT group (5.6 % vs. 5.0 %; OR = 1.15, 95 % CI 0.97–1.37, *p* = 0.11; Fig. 3B). The sICH rates of the adjusted effect estimates indicated a possible higher risk of sICH was achieved for the BT group (OR = 1.20, 95 % CI 1.00–1.44, *p* = 0.05; Fig. S2B in the Supplementary File).

### Secondary efficacy outcomes

**Fig. 4:**
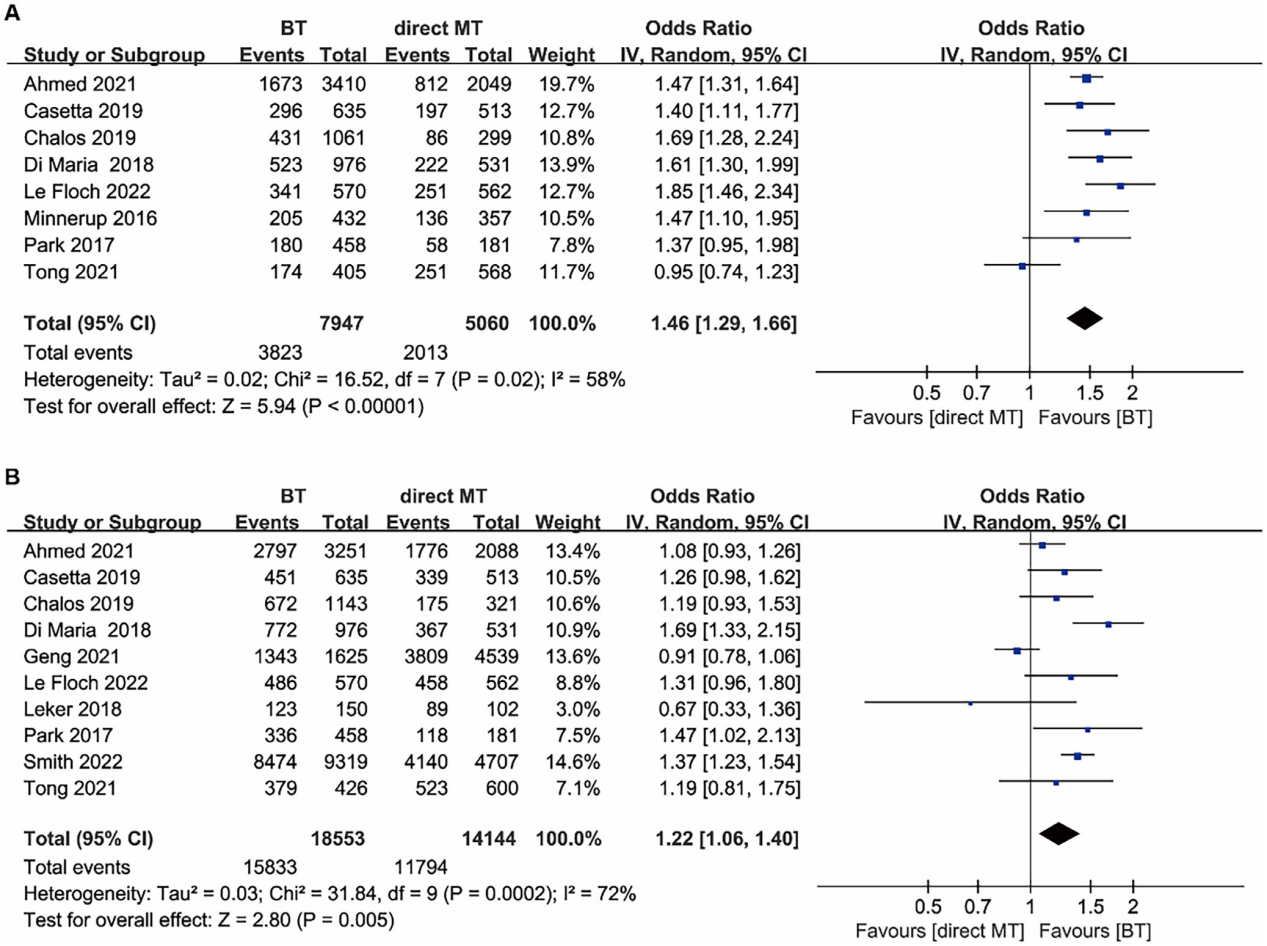
Forest plot of the odds ratios of the secondary outcomes in patients with acute ischaemic stroke: (A) Favourable functional outcome (mRS score 0–2) at 90 days; (B) Successful reperfusion. BT, Bridging Therapy; CI, Confidence Interval; IV, Inverse Variance; MT, Mechanical Thrombectomy; mRS, Modified Rankin Scale.

For the favorable functional outcome (mRS score 0–2) at 90 days, the BT group was superior to the direct MT group (OR = 1.46, 95 % CI 1.29–1.66, *p* < 0.00001; Fig. 4A), with moderate heterogeneity (I^2^ = 58 %). The BT group also had higher odds of successful reperfusion on post-procedural angiography (Thrombolysis in Cerebral Infarction Score ≥ 2b) (OR = 1.22, 95 % CI 1.06–1.40, *p* = 0.005; Fig. 4B), with moderate heterogeneity (I^2^ = 72 %).

### Sensitivity analysis

The sensitivity analysis, by limiting the studies to those on acute ischaemic stroke attributable to anterior circulation occlusion, yielded similar results to the overall analyses of the primary efficacy and safety outcomes (Fig. 3A‒D and Table S3 in the Supplementary Files). Compared with the direct MT group, the BT group had significantly higher odds of excellent functional outcome at 90 days (OR = 1.59, 95 % CI 1.45–1.75, *p* < 0.00001; Fig. S3A in the Supplementary File) and favorable discharge disposition (OR = 1.32, 95 % CI 1.27–1.38, *p* < 0.00001; Fig. S3B in the Supplementary File), lower mortality at 90 days (OR = 0.60, 95 % CI 0.53–0.67, *p* < 0.00001; Fig. S3C in the Supplementary File), and no significant difference in the sICH (OR = 1.18, 95 % CI 0.98–1.41, *p* = 0.07; Fig. S3D in the Supplementary File).

## Discussion

The present meta-analysis of real-world data showed that, compared with the direct MT group, the BT group had higher odds of achieving excellent functional outcomes at 90 days, favorable discharge disposition, favorable functional outcomes at 90 days, successful reperfusion outcomes, and decreased mortality at 90 days in patients with AIS due to LVO. The BT group did not show a significant increase in the risk of experiencing sICH in the crude analysis, however, a potential higher risk of sICH was observed in the adjusted analysis. In addition, the present findings were similar to those of the sensitivity analysis of patients with AIS due to anterior circulation occlusion.

RCTs have been performed to evaluate the efficacy and safety of BT compared with direct MT in the past years. Two RCTs, DIRECT-MT and DEVT, were conducted on Chinese individuals. They showed that direct MT was non-inferior to BT with regard to the better functional outcome at 90 days.^34^^,^^35^ The SKIP was conducted in Japan, which failed to demonstrate the noninferiority of MT alone compared with combined IVT plus MT with regard to favorable functional outcome, however, the point estimates of treatment effect for MT alone was nominally slightly better, not worse, compared with combined therapy.^36^ Conversely, two RCTs in Europe showed neither superiority nor non-inferiority.^37^^,^^38^ Recently, the DIRECT-SAFE recruited patients from Asian (China and Vietnam) versus non-Asian (Australia and New Zealand) regions and did not show non-inferiority of direct MT compared with BT.^39^ There is no consensus to guide clinical practice for the superiority of either approach, as these RCTs produced conflicting results. Hence the nationwide registry-based assessment of real-world outcomes represents a meaningful and topical contribution to the growing body of literature on this topic. The present study included real-world data from multiple large-sample nationwide registry databases that incorporated Asian and non-Asian populations, and it showed that BT was associated with better functional outcomes in patients with AIS due to LVO. Therefore, these findings may provide valuable additional information concerning BT versus direct MT in patients with AIS due to LVO.

Previous meta-analyses of the first three RCTs (DEVT, DIRECT-MT and SKIP) suggested among patients with AIS due to LVO, direct MT was non-inferior to BT.^13^^,^^40^, and similar findings were observed in the pooled analysis of the first four trials (DEVT, DIRECT-MT, SKIP, and MR CLEAN—NO IV).^11^^,^^12^^,^^14^ Li et al.’s meta-analysis also found that direct MT had comparable consequences to BT in efficacy and safety outcomes for patients with AIS due to LVO.^4^ Unlike the above studies, the present meta-analysis of real-world data found that BT is more beneficial than direct MT in patients with AIS due to LVO, notably for anterior circulation occlusion. However, the DIRECT-MT and DEVT were performed in a Chinese population. This population has a high prevalence of intracranial large-artery atherosclerotic disease, which is a lower response to IVT than cardioembolic stroke.^41^^,^^42^ Moreover, the authors of DIRECT-MT and DEVT trials also indicated given the relatively long times from hospital arrival to alteplase initiation in these trials, it remains possible that IV alteplase might still be beneficial in the presence of faster delivery times. The SKIP was conducted in Japan, where a low dose of alteplase was used.^36^ These factors could have understated the benefits of alteplase in patients receiving BT. In addition, some RCTs and meta-analyses did not meet the pre-specified noninferiority margins, and it is not impossible that the treatment effect of direct MT may be overestimated. In comparison, this study included real-world data from multiple large-sample nationwide and health insurance claims databases that incorporated Asian and non-Asian populations. In order to provide more comprehensive evidence of efficacy for BT, the authors also assessed the outcomes of routine discharge (to home with or without services). Furthermore, two recent meta-analyses concerning all 6 RCTs indicate that executing MT without the use of alteplase led to worse disability outcomes at the 90 day mark, however, this difference was not statistically significant.^10^^,^^43^ Utilizing this data, the European Stroke Organization-European Society for Minimally Invasive Neurological Therapy (ESO-ESMINT) recommended, on the basis of moderate-quality evidence, that alteplase continue to be used in eligible patients undergoing MT.^43^ A recent individual participant data meta-analysis of RCTs also did not establish non-inferiority of direct MT compared with BT, and differences in clinical outcomes were small between the two groups.^15^ However, the main limitation of the study is that the settings and inclusion criteria of the RCTs underpinning the meta-analysis inherently introduce selection bias. This means that the results are only applicable to patients who present directly to healthcare centers that have the capability of providing endovascular treatment. The findings of the study may not necessarily apply to patients who initially presented to primary stroke centers.^44^. The present findings from real-world data of multiple large-sample nationwide registries support the ESO-ESMINT recommendations and strengthen their validity.

One meta-analysis of RCTs and observational studies with 36,123 patients suggested IVT+MT has slightly higher rates of survival, successful and complete recanalization, and favorable functional outcomes as compared with direct MT, but the raw data of observational studies were used to analyze instead of adjusted effect size for confounding factors^45^ Another meta-analysis including patients with anterior circulation occlusion also showed the odds for functional independence, successful reperfusion, and mortality for combined RCTs and observational studies data favored the use of BT over direct MT.^46^ Recently, pooled analysis of 55 eligible studies (9 RCTs and 46 observational studies) showed that BT did improve the prognosis for AIS patients and did not increase the risk of hemorrhagic transformation compared with direct MT.^47^ The present findings were consistent with these aforementioned meta-analyses (including RCTs and observational studies). However, in observational studies, baseline characteristics may vary between groups, consequently affecting the outcomes. To reduce possible imbalances between baseline characteristics, the authors combined the statistically adjusted OR results from multiple regression and multivariate match analyses. To ensure that the highest quality data sets are included.^16^ only nationwide or health organization registry databases were eligible in the present study. Additionally, the latest, large-sample, nationwide registries have been included in the present study, such as the one maintained by the American Heart Association and American Stroke Association (16,357 patients)^33^ and another by the China Stroke Prevention Project Committee (7674 patients).^29^. As a result, this study incorporated the latest real-world data and benefited from a larger sample size (86,695 patients). Moreover, discharge-modified Rankin Scale score and non-home discharge disposition are good individual predictors of prognostic benefit for patients with AIS following treatment.^48^ In order to provide more comprehensive evidence of efficacy for BT, the authors also assessed the outcomes of routine discharge (to home with or without services).

Although the rate of sICH was slightly higher in patients who underwent BT than in those who underwent direct MT (5.6 % vs. 4.9 %) in the present study, it was still slightly lower than that of previous observational studies on BT (5.8 % in Lin et al., 6.4 % in Trifan et al. and 6.6 % in Ghaith et al.).^11^^,^^12^^,^^45^^,^^46^ In addition, the real-world data showed that there was no statistically significant difference between the BT and direct MT groups in the rate of sICH in the crude analysis. The present results are consistent with those of previous meta-analyses.^11^^,^^12^^,^^45^^,^^46^ which showed that the sICH levels were comparable between the two groups. A recent meta-analysis including six recent RCTs also indicated moderate-certainty evidence suggesting that there is possibly a slight increase in sICH with BT compared with direct MT (5.7% vs. 4.3 %),^10^ but no significant statistical difference between the two groups. Taken together, a slightly increased risk of sICH in patients undergoing BT was observed compared with direct MT in the present study, but no significant statistical difference was observed between the two groups. These findings are consistent with those of previous meta-analyses. Furthermore, there was no significant difference in the incidence of sICH between the two groups when the authors limited the analysis to patients with AIS due to anterior circulation occlusion. Some studies have shown that IVT may increase the risk of intracranial hemorrhage.^49^ and sICH is associated with poor 90-day functional independence and higher mortality rates. However, these findings indicated that BT had significantly higher odds for functional outcomes at 90 days and discharge disposition outcomes, and it decreased the risk of mortality at 90 days, despite a slightly increased risk of sICH in patients who underwent BT.

The present study has some limitations. First, although the present meta-analysis had a large sample size and data were derived from nationwide or health insurance claims databases, it also reported an adjusted effect size for confounding factors. However, it is limited by potential unmeasured residual confounding factors, such as selective bias, lack of information about patient characteristics, and varied MT strategies, which may explain the heterogeneity identified for several outcomes and potentially influence the reported results. Second, door-to-groin puncture time was not assessed in this meta-analysis owing to the lack of relevant information in most studies. Nevertheless, when the authors have direct evidence to inform patients of important outcomes, surrogate outcomes (for example, door-groin puncture time, successful reperfusion, and any intracranial hemorrhage) are less important.^10^^,^^50^ In addition, the authors could not conduct the subgroup analysis of geographical region (Asian vs. non-Asian regions) because of only two studies recruited patients from the Asian region and provided functional outcomes. Thus, this topic should be explored by more future trials. Third, various criteria were used to define sICH in eligible studies, which might potentially influence the reported results. However, the lack of statistical heterogeneity in the pooled estimates of sICH suggests that these results are valid. Finally, alteplase was primarily used as a thrombolytic agent in the included studies. Therefore, Tenecteplase may be an effective thrombolytic agent. In such cases, additional studies are necessary to determine whether Tenecteplase and MT are more effective than direct MT alone.

## Conclusion

The present meta-analysis of real-world data indicated that in patients with AIS due to LVO, BT was associated with better excellent and favorable functional outcomes at 90 days, favorable discharge disposition, successful reperfusion, and mortality at 90 days, without significantly increasing the risk of sICH. These findings support the current practice in a real-world setting and hence strengthen their validity. For patients with AIS due to LVO who are eligible for both IVT and MT, BT will remain the standard treatment until more data are available.

## Funding

This research did not receive any specific grant from funding agencies in the public, commercial, or not-for-profit sectors.

## CRediT authorship contribution statement

**Bin Qin:** Conceptualization, Investigation, Formal analysis, Methodology, Writing – original draft. **Tao Wei:** Investigation, Formal analysis, Methodology, Writing – review & editing. **Wen Gao:** Investigation, Formal analysis, Methodology, Writing – review & editing. **Hui-xun Qin:** Resources, Writing – review & editing. **Yu-Ming Liang:** Writing – review & editing. **Cheng Qin:** Writing – review & editing. **Hong Chen:** Writing – review & editing. **Ming-Xiu Yang:** Conceptualization, Writing – review & editing, Supervision.

## Declaration of competing interest

The authors declare no conflicts of interest.

## Appendix Supplementary materials

Image, application 1
