# Supplementary material for: Real-world setting comparison of bridging therapy versus direct mechanical thrombectomy for acute ischemic stroke: A meta-analysis
Source: Clinics (Sao Paulo). 2024 May 30;79:100394. doi: 10.1016/j.clinsp.2024.100394 (PMC11177057; doi:10.1016/j.clinsp.2024.100394)

**CLINICS-D-23-00584_Supplementary Material**

**Table Supplementary 1** Search strategy in PubMed database.

| **Search number** | **Search Details** |
| --- | --- |
| 1 | Stroke[Title/Abstract] OR "cerebral ischemia"[Title/Abstract] OR "cerebrovascular ischemia"[Title/Abstract] OR "brain ischemia"[Title/Abstract] OR "intracranial ischemia"[Title/Abstract] OR "cerebral infarct"[Title/Abstract] OR "cerebrovascular infarct"[Title/Abstract] OR "brain infarct"[Title/Abstract] OR "intracranial infarct"[Title/Abstract] OR "cerebral vessel occlusion"[Title/Abstract] OR "large vessel occlusion"[Title/Abstract] OR "cerebrovascular occlusion"[Title/Abstract] OR "intracranial occlusion"[Title/Abstract] |
| 2 | Thrombectomy[Title/Abstract] OR thrombectom*[Title/Abstract] OR mechanical[Title/Abstract] OR endovascular[Title/Abstract] OR embolectomy[Title/Abstract] OR intracranial intervention[Title/Abstract] OR stent-retriever[Title/Abstract] OR solitaire[Title/Abstract] OR trevo[Title/Abstract] OR aspiration[Title/Abstract] OR Penumbra[Title/Abstract] |
| 3 | bridging*[Title/Abstract] OR thrombolysis[Title/Abstract] OR rtPA[Title/Abstract] OR tpA[Title/Abstract] OR rt PA[Title/Abstract] OR alteplase[Title/Abstract] OR plasminogen activator[Title/Abstract] OR recombinant plasminogen[Title/Abstract] OR plasminogen-activator[Title/Abstract] |
| 4 | direct[Title/Abstract] OR alone[Title/Abstract] OR stand-alone[Title/Abstract] OR combined[Title/Abstract] OR with[Title/Abstract] OR combination[Title/Abstract] OR concomitant[Title/Abstract] OR together[Title/Abstract] OR preceding[Title/Abstract] OR pre interventional[Title/Abstract] OR prior[Title/Abstract] OR before[Title/Abstract] OR previous[Title/Abstract] OR following[Title/Abstract] OR followed[Title/Abstract] OR preproced*[Title/Abstract] OR preinterv*[Title/Abstract] OR prethrom*[Title/Abstract] OR pre-proced*[Title/Abstract] OR preinter*[Title/Abstract] OR pre-throm*[Title/Abstract] |
|  | real world[Title/Abstract] OR observational[Title/Abstract] OR registry[Title/Abstract] OR cohort[Title/Abstract] OR retrospective[Title/Abstract] OR prospective[Title/Abstract] |
| 5 | 1 AND 2 AND 3 AND 4 |

**Table Supplementary 2** Risk of bias assessment in the included studies using Risk of Bias in Non-randomized Studies of Interventions (ROBINS-I) tool.

| **Study/ Bias domain** | **Bias due to confounding** | **Bias in selection of participants into the study** | **Bias in classification of interventions** | **Bias due to deviations from intended interventions** | **Bias due to missing data** | **Bias in measurement of outcomes** | **Bias in selection of the reported result** | **Overall** |
| --- | --- | --- | --- | --- | --- | --- | --- | --- |
| Ahmed 2021 | Low risk of bias | Low risk of bias | Low risk of bias | Low risk of bias | Low risk of bias | Low risk of bias | Low risk of bias | Low risk of bias |
| Casetta 2019 | Low risk of bias | Low risk of bias | Low risk of bias | Low risk of bias | No Information | Low risk of bias | Low risk of bias | Moderate risk of bias |
| Chalos 2019 | Low risk of bias | Low risk of bias | Low risk of bias | Low risk of bias | Low risk of bias | Low risk of bias | Low risk of bias | Low risk of bias |
| Di Maria 2018 | Low risk of bias | Low risk of bias | Low risk of bias | Low risk of bias | Low risk of bias | Low risk of bias | Low risk of bias | Low risk of bias |
| Dicpinigaitis 2022 | Moderate risk of bias Multivariable logistic regression analysis for certain important confounders, but there may still be variables unbalanced. | Low risk of bias | Low risk of bias | Low risk of bias | No Information | Low risk of bias | Low risk of bias | Moderate risk of bias |
| Geng 2021 | Low risk of bias | Low risk of bias | Low risk of bias | Low risk of bias | No Information | Low risk of bias | Low risk of bias | Moderate risk of bias |
| Le Floch 2022 | Low risk of bias | Low risk of bias | Low risk of bias | Low risk of bias | Low risk of bias | Low risk of bias | Low risk of bias | Low risk of bias |
| Leker 2018 | Moderate risk of bias Multivariable logistic regression analysis for certain important confounders, but there may still be variables unbalanced. | Low risk of bias | Low risk of bias | Low risk of bias | No Information | Low risk of bias | Low risk of bias | Moderate risk of bias |
| Minnerup 2016 | Low risk of bias | Low risk of bias | Low risk of bias | Low risk of bias | Low risk of bias | Low risk of bias | Low risk of bias | Low risk of bias |
| Park 2017 | Low risk of bias | Low risk of bias | Low risk of bias | Low risk of bias | Low risk of bias | Low risk of bias | Low risk of bias | Low risk of bias |
| Smith 2022 | Low risk of bias | Low risk of bias | Low risk of bias | Low risk of bias | Low risk of bias | Low risk of bias | Low risk of bias | Low risk of bias |
| Tong 2021 | Low risk of bias | Low risk of bias | Low risk of bias | Low risk of bias | No Information | Low risk of bias | Low risk of bias | Moderate risk of bias |

**Table Supplementary 3** Sensitivity analysis of main outcomes by limiting the studies to those on acute ischemic stroke attributable to anterior circulation occlusion (Bridging therapy vs. Direct Mechanical thrombectomy).

| **Outcomes** | **Anterior circulation large vessel occlusion stroke** | **All Patients** |
| --- | --- | --- |
| **Primary Efficacy Outcomes** |  |  |
| Excellent functional outcome at 90 days | OR=1.59, 95% CI (1.45–1.75), p<0.00001 | OR=1.48, 95% CI (1.25–1.75), p<0.00001 |
| Favorable discharge disposition (to home with or without services) | OR=1.32, 95% CI (1.27–1.38), p<0.00001 | OR=1.33, 95% CI (1.29–1.38), p<0.00001 |
| **Safety Outcomes** |  |  |
| Mortality at 90 days | OR=0.60, 95% CI (0.53–0.67), p<0.00001 | OR=0.62, 95% CI (0.56–0.70), p<0.00001 |
| Symptomatic intracranial haemorrhage | OR=1.18, 95% CI (0.98–1.41), p=0.07 | OR=1.15, 95% CI (0.97–1.37), p=0.11 |

CI, Confidence Interval; OR, Odds Ratio.

**Figure Supplementary 1** Forest plot of the adjusted odds ratios of the primary efficacy outcomes in patients with acute ischemic stroke: (A) Excellent functional outcome (mRS 0–1) at 90 days; (B) Favorable discharge disposition (to home with or without services). BT, Bridging Therapy; CI, Confidence Interval; IV, Inverse Variance; MT, Mechanical Thrombectomy; mRS, Modified Rankin Scale; SE, Standard Error.


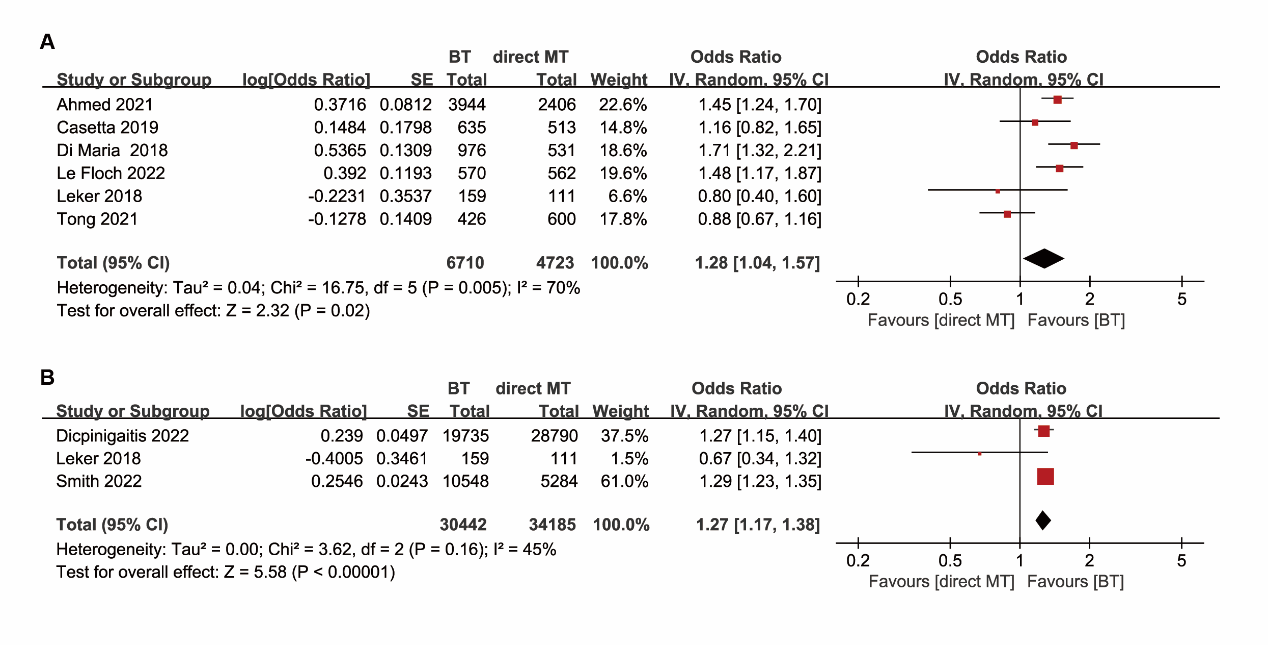


**Figure Supplementary 2** Forest plot of the adjusted odds ratios of the safety outcomes in patients with acute ischemic stroke: (A) Mortality at 90 days; (B) Symptomatic intracranial hemorrhage. BT, Bridging Therapy; CI, Confidence Interval; IV, Inverse Variance; MT, Mechanical Thrombectomy; SE, Standard Error.


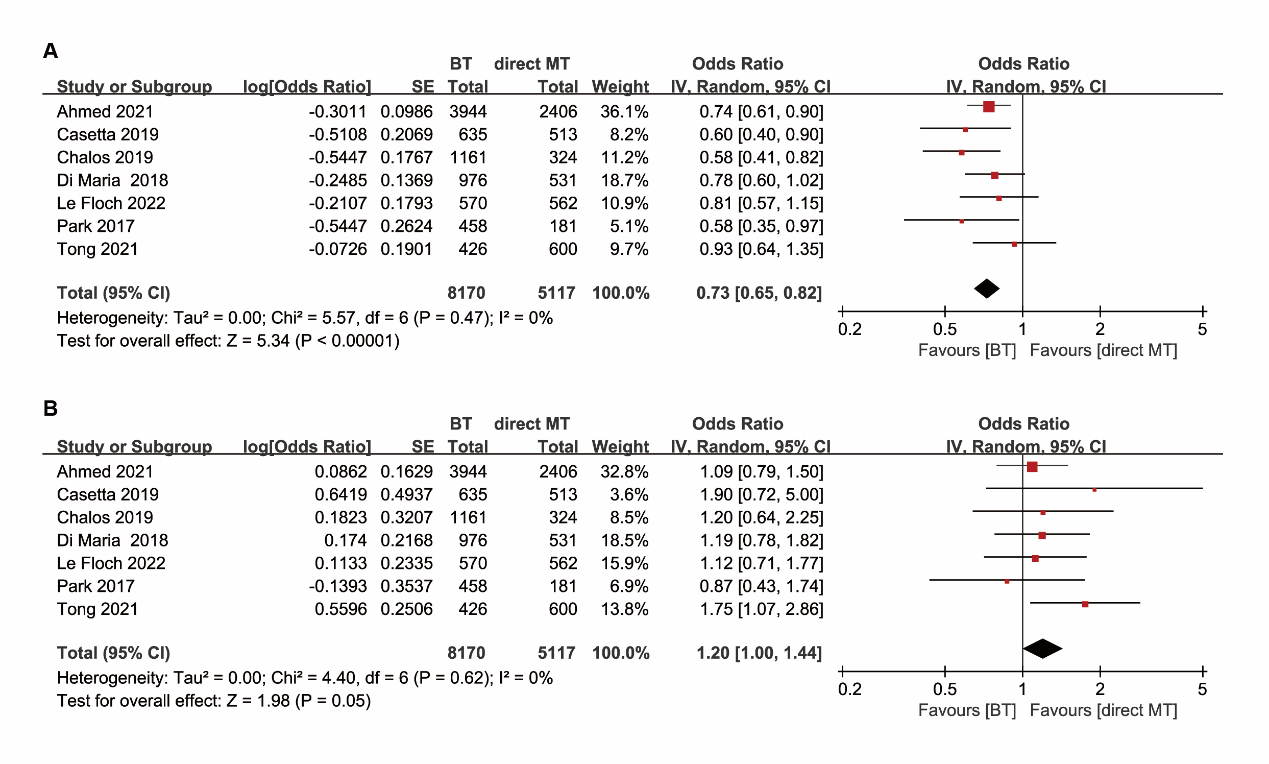


**Figure Supplementary 3** Forest plot of the sensitivity analysis by limiting the studies to those on acute ischemic stroke attributable to anterior circulation occlusion: (A) Excellent functional outcome (mRS 0–1) at 90 days; (B) Favorable discharge disposition (to home with or without services); (C) Mortality at 90 days; (D) Symptomatic intracranial hemorrhage. BT, Bridging Therapy; CI, Confidence Interval; IV, Inverse Variance; MT, Mechanical Thrombectomy; mRS, Modified Rankin Scale; SE, Standard Error.


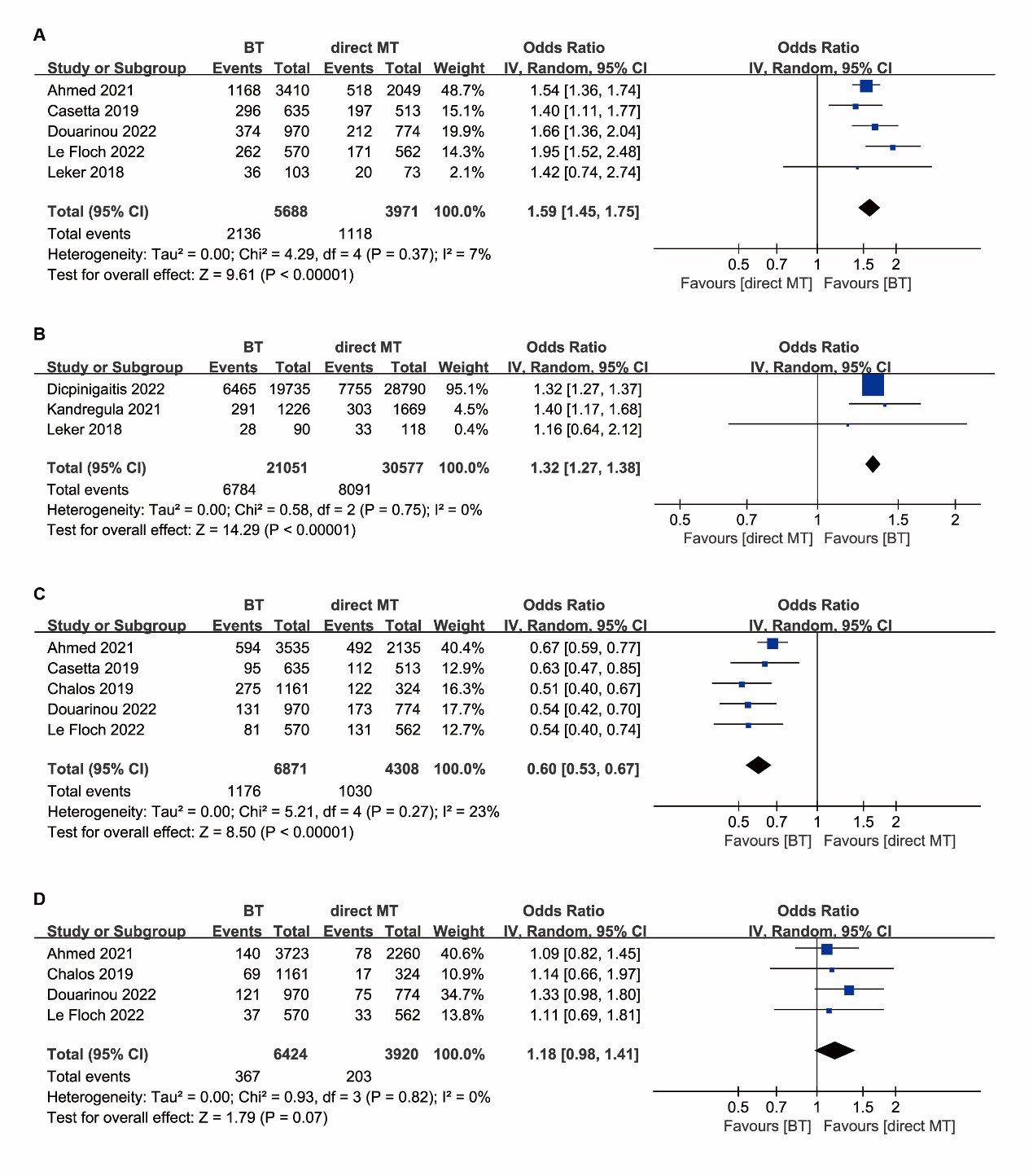

Supplement: Supplementary file 1 [file mmc1.docx]
